# National Baby Week

**Published:** 1918-07-06

**Authors:** 


### NATIONAL BABY WEEK.

### The Council's Appeal.

We received too late for insertion in our last issue a letter signed by the Chairman, Chairman of Executive, Honorary Secretary, and Honorary Treasurer of the National Baby Week Council, which states that the results of last year's work cannot be estimated in any known terms, but more than 600 local committees were organised. This year the Council hopes to see the number of local committees at least doubled, and they ask mayors and other municipal authorities to organise public meetings, clergymen to preach sermons, and teachers in schools to give lessons, so that the facts as to infant mortality and the means by which it can be prevented may be poured into every ear that is capable of hearing and understanding.

### At Central Hall, Westminster.

The Educational Mothercraft Exhibition, National Baby Week, July 1-6, included a model ward for ailing babies, with baby dolls dressed in suitable clothing; a specimen model infant welfare centre, and specimen home, where the mother is supposed to be carrying out the teaching received at the centre, model village homes, housing exhibits, plans and photos of garden suburbs.

The Duchess of Marlborough spoke on the Duties of Women as Municipal Electors at the mass meeting on Monday, when the Bishop of Birmingham discussed Environment as Affecting the Mother and Infant, Mrs. H. B. Irving Mothers' Pensions, and Mr. Ben Tillett the State and the Expectant Mother.

